# Comorbidity burden and outcomes of endoscopic ultrasound‐guided treatment of pancreatic fluid collections: Multicenter study with nationwide data‐based validation

**DOI:** 10.1111/den.14924

**Published:** 2024-09-26

**Authors:** Tsuyoshi Hamada, Atsuhiro Masuda, Nobuaki Michihata, Tomotaka Saito, Masahiro Tsujimae, Mamoru Takenaka, Shunsuke Omoto, Takuji Iwashita, Shinya Uemura, Shogo Ota, Hideyuki Shiomi, Toshio Fujisawa, Sho Takahashi, Saburo Matsubara, Kentaro Suda, Hiroki Matsui, Akinori Maruta, Kensaku Yoshida, Keisuke Iwata, Mitsuru Okuno, Nobuhiko Hayashi, Tsuyoshi Mukai, Kiyohide Fushimi, Ichiro Yasuda, Hiroyuki Isayama, Hideo Yasunaga, Yousuke Nakai, the WONDERFUL study group in Japan and collaborators, Tatsuya Sato, Arata Sakai, Ryota Nakano, Keito Nakagawa, Yuhei Iwasa

**Affiliations:** ^1^ Department of Gastroenterology, Graduate School of Medicine The University of Tokyo Tokyo Japan; ^2^ Department of Health Services Research, Graduate School of Medicine The University of Tokyo Tokyo Japan; ^3^ Department of Clinical Epidemiology and Health Economics, School of Public Health The University of Tokyo Tokyo Japan; ^4^ Department of Hepato‐Biliary‐Pancreatic Medicine, Cancer Institute Hospital Japanese Foundation for Cancer Research Tokyo Japan; ^5^ Department of Gastroenterology, Graduate School of Medicine Juntendo University Tokyo Japan; ^6^ Department of Health Policy and Informatics, Graduate School of Medical and Dental Sciences Tokyo Medical and Dental University Tokyo Japan; ^7^ Department of Endoscopy and Endoscopic Surgery The University of Tokyo Hospital Tokyo Japan; ^8^ Division of Gastroenterology, Department of Internal Medicine Kobe University Graduate School of Medicine Hyogo Japan; ^9^ Division of Hepatobiliary and Pancreatic Diseases, Department of Gastroenterology Hyogo Medical University Hyogo Japan; ^10^ Department of Gastroenterology and Hepatology, Faculty of Medicine Kindai University Osaka Japan; ^11^ First Department of Internal Medicine Gifu University Hospital Gifu Japan; ^12^ Department of Gastroenterology Gifu Prefectural General Medical Center Gifu Japan; ^13^ Department of Gastroenterology Gifu Municipal Hospital Gifu Japan; ^14^ Department of Gastroenterology and Hepatology, Saitama Medical Center Saitama Medical University Saitama Japan; ^15^ Third Department of Internal Medicine University of Toyama Toyama Japan; ^16^ Department of Gastroenterological Endoscopy Kanazawa Medical University Ishikawa Japan

**Keywords:** acute necrotizing pancreatitis, cohort study, endosonography, risk factor, stent

## Abstract

**Objectives:**

The appropriate holistic management is mandatory for successful endoscopic ultrasound (EUS)‐guided treatment of pancreatic fluid collections (PFCs). However, comorbidity status has not been fully examined in relation to clinical outcomes of this treatment.

**Methods:**

Using a multi‐institutional cohort of 406 patients receiving EUS‐guided treatment of PFCs in 2010–2020, we examined the associations of Charlson Comorbidity Index (CCI) with in‐hospital mortality and other clinical outcomes. Multivariable logistic regression analysis was conducted with adjustment for potential confounders. The findings were validated using a Japanese nationwide inpatient database including 4053 patients treated at 486 hospitals in 2010–2020.

**Results:**

In the clinical multi‐institutional cohort, CCI was positively associated with the risk of in‐hospital mortality (*P*_trend_ < 0.001). Compared to patients with CCI = 0, patients with CCI of 1–2, 3–5, and ≥6 had adjusted odds ratios (95% confidence intervals) of 0.76 (0.22–2.54), 5.39 (1.74–16.7), and 8.77 (2.36–32.6), respectively. In the nationwide validation cohort, a similar positive association was observed; the corresponding odds ratios (95% confidence interval) were 1.21 (0.90–1.64), 1.52 (0.92–2.49), and 4.84 (2.63–8.88), respectively (*P*_trend_ < 0.001). The association of higher CCI with longer length of stay was observed in the nationwide cohort (*P*_trend_ < 0.001), but not in the clinical cohort (*P*_trend_ = 0.18). CCI was not associated with the risk of procedure‐related adverse events.

**Conclusions:**

Higher levels of CCI were associated with a higher risk of in‐hospital mortality among patients receiving EUS‐guided treatment of PFCs, suggesting the potential of CCI in stratifying the periprocedural mortality risk.

**Trial registration:**

The research based on the clinical data from the WONDERFUL cohort was registered with UMIN‐CTR (registration number UMIN000044130).

## INTRODUCTION

Pancreatic fluid collections (PFCs) mainly consist of walled‐off necrosis and a pancreatic pseudocyst, which occur as local complications of acute pancreatitis.^1^, ^2^ When the symptoms of PFCs become refractory to conservative management, patients are usually hospitalized and referred to drainage‐based interventions.^3^ With the increasing popularity of endoscopic ultrasound (EUS)‐guided transluminal interventions, endoscopic procedures currently serve as a first‐line treatment option for symptomatic PFCs at many centers.^4^, ^5^, ^6^ The emerging treatment modality of lumen‐apposing metal stents (LAMS) has revolutionized nonsurgical management by serving as a transmural port for endoscopic necrosectomy following EUS‐guided drainage of PFCs.^7^, ^8^, ^9^, ^10^, ^11^ Despite the increasing safety and effectiveness of the EUS‐guided treatment, a substantial proportion of patients undergo procedure‐related adverse events and fatal outcomes^12^, ^13^, ^14^ because of the invasiveness of the procedure and poor conditions of the patients treated. Given the high risk of procedure‐related mortality, it is important to identify predictive factors for clinical outcomes of PFCs treated by EUS‐guided drainage and adjunctive treatment. To date, risk stratification has been attempted predominantly based on the information on characteristics of PFCs (e.g., the types of PFCs, the proportion of necrosis, PFC extension status) and procedures (e.g., necrosectomy).^14^, ^15^, ^16^, ^17^

A better understanding of patient characteristics associated with adverse outcomes of EUS‐guided PFC treatment would facilitate clinical decision‐making and help optimize the treatment algorithm for patients receiving this treatment. Comorbidity burden has been implicated in the risk of long‐term mortality in different populations, including patients presenting with severe acute conditions and those diagnosed with cancer.^18^, ^19^, ^20^ Charlson Comorbidity Index (CCI) has been widely utilized to assess the levels of comorbid conditions of individuals in clinical research based on the information on comorbidities (e.g., diabetes, cardio‐ and cerebrovascular diseases).^20^ In the field of pancreatology, studies have linked CCI to clinical outcomes of patients presenting with acute pancreatitis and those receiving pancreatic surgery.^21^, ^22^, ^23^, ^24^, ^25^ Given the wide applicability of CCI to the mortality prediction for acute severe diseases, we hypothesized that higher levels of CCI might be associated with a higher risk of in‐hospital mortality in patients receiving EUS‐guided treatment of PFCs.

To test our hypothesis, we utilized a clinical multi‐institutional cohort of patients receiving EUS‐guided PFC treatment, which was established by the WONDERFUL (WON anD pERipancreatic FlUid coLlection) study group,^26^, ^27^ and validated our findings in a Japanese nationwide inpatient database (the Diagnosis Procedure Combination [DPC] database).

## METHODS

### Study design

**Figure 1:**
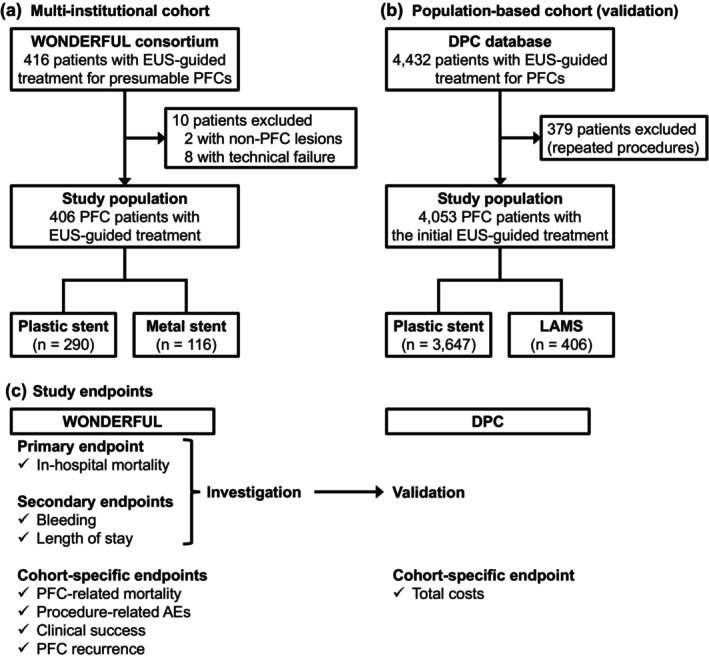
Study outline. (a) Flow diagram of selection of patients receiving endoscopic ultrasound (EUS)‐guided treatment of pancreatic fluid collections (PFCs) in a clinical multi‐institutional cohort within the WONDERFUL consortium. (b) Flow diagram of selection of patients receiving EUS‐guided treatment of PFCs in a population‐based cohort within the Diagnosis Procedure Combination (DPC) database. (c) Study end‐points. The primary and secondary end‐points according to Charlson Comorbidity Index were examined in the WONDERFUL cohort, and the findings were validated in the DPC cohort. Relevant end‐points that were assessable only in either cohort were additionally examined. AE, adverse event; LAMS, lumen‐apposing metal stent.

This retrospective cohort study aimed to examine the associations of CCI with clinical outcomes of patients receiving EUS‐guided PFC treatment (Fig. 1). We conducted the primary analyses using a clinical multi‐institutional cohort (WONDERFUL cohort) and validated our findings using Japanese nationwide inpatient data (DPC cohort) when study end‐points were available. The primary end‐point of this study was all‐cause in‐hospital mortality. Secondary end‐points included bleeding and length of stay. Cohort‐specific end‐points were PFC‐related mortality, all procedure‐related adverse events (e.g., bleeding, perforation), clinical success, and PFC recurrence in the WONDERFUL cohort; and total costs calculated in 2021 US dollars in the DPC cohort.

The current study was undertaken according to the guidelines in the Helsinki Declaration.^28^ In the WONDERFUL cohort, the study was approved by the ethics committee at each center and was registered with UMIN‐CTR (registration number UMIN000044130). Written informed consent for the procedure was obtained from all patients, and consent for the use of the retrospective data for research was obtained on an opt‐out basis. The use of the DPC database for the current study was approved by the institutional review board of The University of Tokyo (Tokyo, Japan). The requirement for patient informed consent was waived because of the anonymous nature of the data used.

### Study population

Within the WONDERFUL consortium of 10 high‐volume centers in Japan (Appendix S1), we identified consecutive patients who received EUS‐guided treatment of PFCs from January 1, 2010, through November 30, 2020 (Fig. 1a). We categorized PFCs into walled‐off necrosis or pancreatic pseudocysts according to the revised Atlanta classification.^2^ We excluded patients with PFCs treated by EUS‐guided aspiration alone. We further excluded patients with technical failure in EUS‐guided drainage and patients with non‐PFC lesions managed via EUS‐guided drainage. Utilizing a standardized study database constructed via the Microsoft Access software (Microsoft Japan, Tokyo, Japan), study physicians reviewed medical charts and collected the clinical data. The patients were followed up until death or the end of follow‐up (May 31, 2023), whichever came first.

Using the DPC database, which is a national administrative database of inpatient care in Japan,^29^, ^30^ we identified consecutive patients who received EUS‐guided treatment of PFCs (coded as the Japanese original code, K7071) between July 1, 2010, and March 31, 2020 (Fig. 1b). This database includes admission/discharge abstract data and administrative claims data from more than 1200 hospitals throughout Japan including all university hospitals. Data on diagnoses, comorbidities present at admission, and complications occurring during hospitalization are coded using the International Classification of Diseases 10th Revision (ICD‐10) codes. The database also contains detailed clinical information including but not limited to age, sex, dates of admission and discharge, body height/weight, intensive care unit admission, discharge status (e.g., in‐hospital death), nonsurgical or surgical procedures (indexed by Japanese original codes), medications, select devices (indexed by specific codes), and medical costs. For patients who underwent multiple hospitalizations associated with EUS‐guided PFC drainage, we included the first procedure performed during the initial hospitalization and excluded subsequent hospitalizations.

### Charlson Comorbidity Index

The scoring system of CCI consists of 19 items corresponding to medical comorbid conditions with different weights according to the potential impact on the risk of long‐term mortality.^31^ CCI is calculated as the summation of the weights with higher scores corresponding to higher levels of comorbidity burden (Table S1). In the WONDERFUL cohort, clinical diagnoses were used to evaluate the comorbidities. In the DPC cohort, ICD‐10 codes recorded in the database were used to identify the comorbidities. In validation studies based on independent populations, the agreement regarding the scoring of CCI has been high between ICD‐10 codes, self‐report, and medical charts.^20^

### Endoscopic ultrasound–guided and adjunctive treatment of PFCs

**Figure 2:**
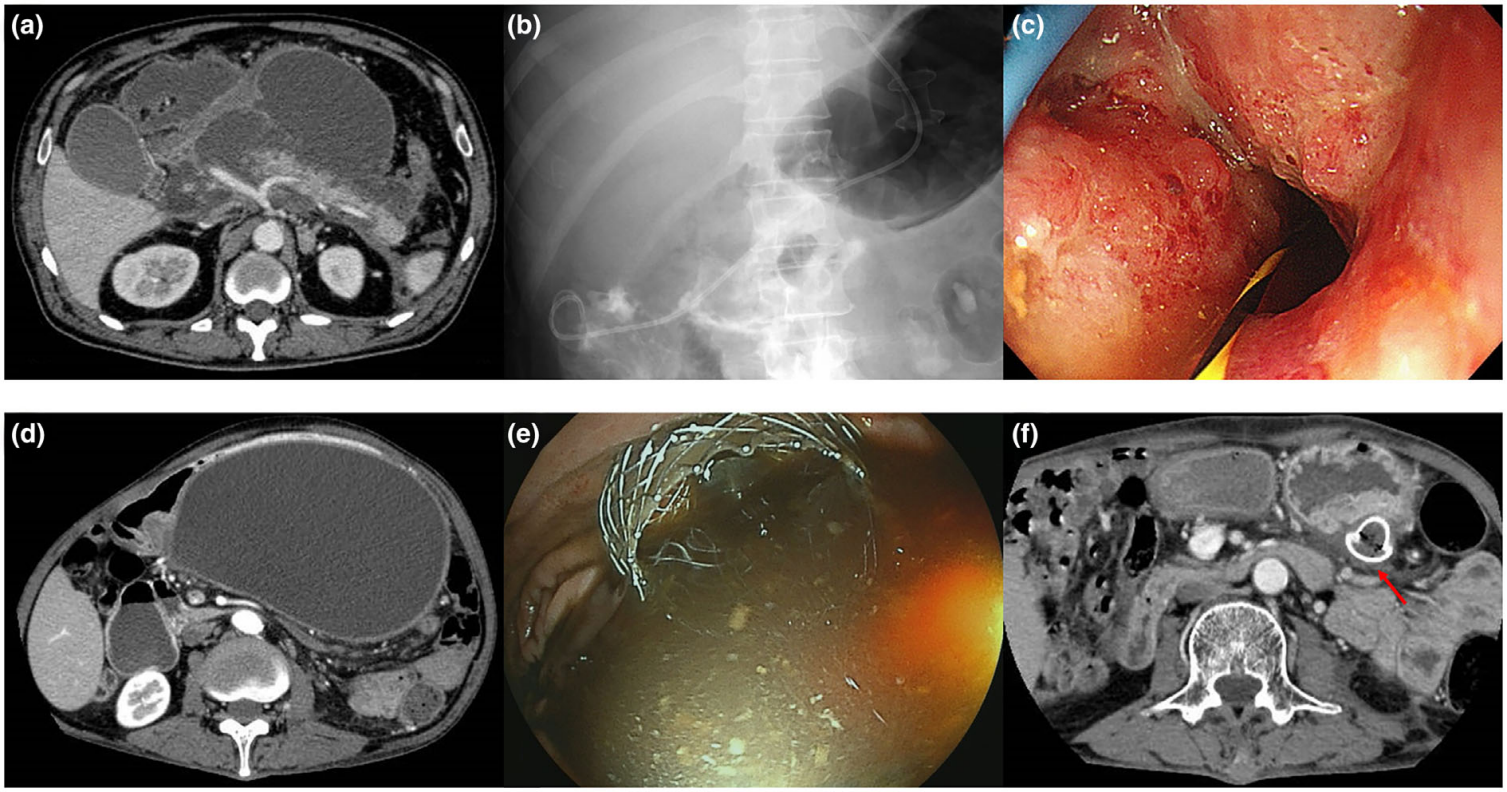
Procedures of endoscopic ultrasound (EUS)‐guided treatment of pancreatic fluid collections in a clinical multi‐institutional cohort within the WONDERFUL consortium (a–c, walled‐off necrosis [WON]; d–f, pseudocyst). (a) Contrast‐enhanced computed tomography delineating large WON extending to the pelvic cavity. (b) Fluoroscopic image of EUS‐guided drainage via the transgastric placement of a lumen‐apposing metal stent (LAMS) and coaxial nasocystic catheter. (c) Endoscopic image showing successful clearance of necrotic contents after four sessions of endoscopic necrosectomy. (d) Contrast‐enhanced computed tomography delineating a large pseudocyst. (e) Endoscopic image after EUS‐guided transgastric placement of a LAMS with a discharge of infectious intracystic fluid. (f) Contrast‐enhanced computed tomography delineating clinical treatment success with a reduction in the size of the pseudocyst (arrow, a LAMS in situ).

The procedures of EUS‐guided and adjunctive treatment in the WONDERFUL cohort are described in Appendix S2 and Figure 2.

### Definitions of covariates and other outcome variables

In the WONDERFUL cohort, a PFC‐related death was defined when a patient died with PFC‐related symptoms (e.g., sepsis), adverse events associated with PFC treatment, or underlying pancreatitis. Procedure‐related adverse events (e.g., bleeding, peritonitis, pancreatitis) were defined and graded according to the American Society for Gastrointestinal Endoscopy lexicon guidelines.^32^ Clinical success was defined as a reduction in the PFC size to ≤2 cm or removal of all transmural and percutaneous stents/catheters with relief of symptoms associated with PFCs. Time to clinical success was defined as the time from the initial EUS‐guided drainage to clinical success. For cases with clinical success, PFC recurrence was defined as the occurrence of a new PFC or exacerbation of the treated PFC on cross‐sectional imaging studies. Time to PFC recurrence was defined as the time from clinical success to PFC recurrence.

In the DPC cohort, bleeding was defined when a hemostatic procedure (via endoscopy, interventional radiology, or surgery) was performed following the initial EUS‐guided drainage. The data on the indications for transfusion were not available in the database, and patients with higher CCI were more likely to be transfused for underlying diseases; therefore, transfusion was not included in the definition of bleeding. Hospital case volume was defined as the average annual number of patients receiving EUS‐guided drainage of PFCs at a given hospital.^29^ Information on the causes of in‐hospital deaths was not available.

### Statistical analysis

A detailed description of the statistical analysis is presented in Appendix S3. Our primary hypothesis testing was an assessment of the association of CCI with the in‐hospital mortality rate of patients receiving EUS‐guided treatment of PFCs in the multivariable logistic regression model. To calculate odds ratios (ORs) and 95% confidence intervals (CIs) for in‐hospital mortality according to CCI, we used the multivariable logistic regression model.

All statistical analyses were performed using the Stata software (version 18; StataCorp LLC, College Station, TX, USA), unless otherwise noted, and all *P*‐values were two‐sided. Given multiple comparisons, we used the stringent two‐sided α level of 0.005 for statistical significance.^33^

## RESULTS

The current study included 406 patients receiving EUS‐guided treatment of PFCs in the WONDERFUL cohort and 4053 patients treated at 486 hospitals (102 academic and 384 community hospitals) in the DPC cohort (Fig. 1, Tables 1, 2). Higher levels of CCI were associated with older age, lower levels of albumin, and hospitalizations at nonacademic hospitals. The in‐hospital mortality was observed in 28 (6.9%) and 228 (5.6%) patients in the WONDERFUL and DPC cohorts, respectively.

**Table 1:** Clinical characteristics of and treatment modalities for patients receiving endoscopic ultrasound (EUS)‐guided treatment of pancreatic fluid collections (PFCs), overall or by Charlson Comorbidity Index (a multi‐institutional cohort within the WONDERFUL consortium)

Characteristics^†^	All cases (*n* = 406)	Charlson Comorbidity Index				*P*‐value
		0 (*n* = 174)	1–2 (*n* = 151)	3–5 (*n* = 52)	≥6 (*n* = 29)	
Mean age ± SD, years	60.4 ± 13.7	56.6 ± 13.7	61.4 ± 13.3	68.5 ± 11.0	63.0 ± 12.9	<0.001
Sex						0.18
Male	311 (77%)	124 (71%)	122 (81%)	42 (81%)	23 (79%)	‐
Female	95 (23%)	50 (29%)	29 (19%)	10 (19%)	6 (21%)	‐
Year of admission						0.36
2010–2013	118 (29%)	54 (31%)	47 (31%)	12 (23%)	5 (17%)	‐
2014–2016	95 (23%)	35 (20%)	38 (25%)	16 (31%)	6 (21%)	‐
2017–2020	193 (48%)	85 (49%)	66 (44%)	24 (46%)	18 (62%)	‐
Body mass index						0.68
<25 kg/m^2^	340 (85%)	141 (83%)	129 (86%)	44 (90%)	26 (90%)	‐
25–29.9 kg/m^2^	47 (12%)	22 (13%)	17 (11%)	5 (10%)	3 (10%)	‐
≥30 kg/m^2^	11 (2.8%)	7 (4.1%)	4 (2.7%)	0 (0.0%)	0 (0.0%)	‐
Preprocedural ICU admission						0.030
No	385 (95%)	159 (91%)	148 (98%)	49 (94%)	29 (100%)	‐
Yes	21 (5.2%)	15 (8.6%)	3 (2.0%)	3 (5.8%)	0 (0.0%)	‐
Preprocedural WBC, 10^9^/L	8.9 (6.2–13.1)	8.4 (6.0–12.8)	8.6 (6.3–13.8)	9.5 (6.5–11.9)	9.8 (7.4–12.7)	0.65
Preprocedural albumin, g/dL	2.8 (2.3–3.3)	2.9 (2.4–3.5)	2.8 (2.2–3.3)	2.6 (2.1–3.0)	2.6 (2.3–2.9)	0.002
Type of PFC						0.072
Walled‐off necrosis	232 (57%)	110 (63%)	85 (57%)	26 (50%)	11 (38%)	‐
Pseudocyst	123 (30%)	49 (28%)	46 (30%)	15 (29%)	13 (45%)	‐
Postoperative PFC	51 (13.0%)	15 (8.6%)	20 (13.0%)	11 (21.0%)	5 (17.0%)	‐
Size of PFC, cm	10.0 (6.9–14.3)	10.0 (6.9–14.8)	9.7 (6.6–14.1)	10.0 (7.0–14.3)	8.1 (6.9–11.7)	0.61
Injury of the pancreatic duct						0.16
Absent	260 (65%)	104 (62%)	101 (67%)	32 (62%)	23 (82%)	‐
DPDS and/or disruption	139 (35%)	65 (38%)	49 (33%)	20 (38%)	5 (18%)	‐
Indication of EUS‐guided drainage						0.47
Infection	258 (64%)	101 (58%)	98 (65%)	35 (67%)	24 (83%)	‐
Abdominal pain	75 (18%)	34 (20%)	29 (19%)	9 (17%)	3 (10%)	‐
Expanding collection	58 (14.0%)	31 (18.0%)	19 (13.0%)	6 (12.0%)	2 (6.9%)	‐
Others	15 (3.7%)	8 (4.6%)	5 (3.3%)	2 (3.8%)	0 (0.0%)	‐
Route of EUS‐guided drainage						0.43
Transgastric	376 (93%)	163 (94%)	142 (94%)	44 (85%)	27 (93%)	‐
Transduodenal	27 (6.7%)	10 (5.7%)	8 (5.3%)	7 (13%)	2 (6.9%)	‐
Transesophageal	3 (0.7%)	1 (0.6%)	1 (0.7%)	1 (1.9%)	0 (0.0%)	‐
Stent type						0.79
Plastic stent	290 (71%)	122 (70%)	108 (72%)	37 (71%)	23 (79%)	‐
Metal stent (including LAMS)	116 (29%)	52 (30%)	43 (28%)	15 (29%)	6 (21%)	‐
Multigateway approach						0.19
Absent	358 (88%)	151 (87%)	130 (86%)	50 (96%)	27 (93%)	‐
Present	48 (12.0%)	23 (13.0%)	21 (14.0%)	2 (3.8%)	2 (6.9%)	‐
Percutaneous drainage						0.90
Absent	375 (92%)	160 (92%)	141 (93%)	47 (90%)	27 (93%)	‐
Present	31 (7.6%)	14 (8.0%)	10 (6.6%)	5 (9.6%)	2 (6.9%)	‐
Endoscopic necrosectomy						0.50
Absent	294 (72%)	120 (69%)	111 (74%)	40 (77%)	23 (79%)	‐
Present	112 (28%)	54 (31%)	40 (26%)	12 (23%)	6 (21%)	‐

^†^ Percentage indicates the proportion of cases with a specific characteristic in all cases or each stratum of Charlson Comorbidity Index. Total percentages may not equal 100% due to rounding.

DPDS, disconnected pancreatic duct syndrome; ICU, intensive care unit; LAMS, lumen‐apposing metal stent; SD, standard deviation; WBC, white blood cell.

[Correction added on 19 October 2024, after first online publication: A ‘0’ at the end of each *P*‐value has been removed.]

**Table 2:** Clinical characteristics of and treatment modalities for patients receiving endoscopic ultrasound‐guided treatment of pancreatic fluid collections, overall or by Charlson Comorbidity Index (a population‐based cohort within the nationwide Diagnosis Procedure Combination [DPC] database)

Characteristics^†^	All cases (*n* = 4053)	Charlson Comorbidity Index				*P*‐value
		0 (*n* = 2125)	1–2 (*n* = 1562)	3–5 (*n* = 276)	≥6 (*n* = 90)	
Mean age ± SD, years	62.8 ± 14.2	61.2 ± 14.7	64.0 ± 13.7	67.7 ± 11.2	63.9 ± 13.0	<0.001
Sex						0.33
Male	2875 (71%)	1494 (70%)	1130 (72%)	192 (70%)	59 (66%)	‐
Female	1178 (29%)	631 (30%)	432 (28%)	84 (30%)	31 (34%)	‐
Year of admission						0.55
2010–2013	1343 (33%)	698 (33%)	534 (34%)	78 (28%)	33 (37%)	‐
2014–2016	1143 (28%)	593 (28%)	439 (28%)	85 (31%)	26 (29%)	‐
2017–2020	1567 (39%)	834 (39%)	589 (38%)	113 (41%)	31 (34%)	‐
Body mass index						0.049
<25 kg/m^2^	3116 (80%)	1643 (81%)	1178 (79%)	212 (80%)	83 (94%)	‐
25–29.9 kg/m^2^	628 (16%)	328 (16%)	255 (17%)	41 (16%)	4 (4.5%)	‐
≥30 kg/m^2^	132 (3.4%)	67 (3.3%)	53 (3.6%)	11 (4.2%)	1 (1.1%)	‐
Preprocedural ICU admission						0.005
No	3758 (93%)	1995 (94%)	1430 (92%)	247 (89%)	86 (96%)	‐
Yes	295 (7.3%)	130 (6.1%)	132 (8.5%)	29 (11%)	4 (4.4%)	‐
Hospital type						<0.001
Nonacademic	1998 (49%)	971 (46%)	812 (52%)	160 (58%)	55 (61%)	‐
Academic	2055 (51%)	1154 (54%)	750 (48%)	116 (42%)	35 (39%)	‐
Hospital case volume						0.004
Q1 (1.0–1.9 per year)	946 (23%)	455 (21%)	387 (25%)	74 (27%)	30 (33%)	‐
Q2 (2.0–3.4 per year)	1015 (25%)	509 (24%)	414 (26%)	72 (26%)	20 (22%)	‐
Q3 (3.5–7.9 per year)	1066 (27%)	584 (28%)	387 (25%)	69 (25%)	26 (29%)	‐
Q4 (≥8.0 per year)	1026 (25%)	577 (27%)	374 (24%)	61 (22%)	14 (16%)	‐
Stent type						0.47
Plastic	3647 (90%)	1898 (89%)	1420 (91%)	248 (90%)	81 (90%)	‐
LAMS	406 (10%)	227 (11%)	142 (9.1%)	28 (10%)	9 (10%)	‐

^†^ Percentage indicates the proportion of cases with a specific characteristic in all cases or each stratum of Charlson Comorbidity Index. Total percentages may not equal 100% due to rounding.

ICU, intensive care unit; LAMS, lumen‐apposing metal stent; Q1–4, quartiles 1–4; SD, standard deviation.

[Correction added on 19 October 2024, after first online publication: A ‘0’ at the end of each *P*‐value has been removed.]

**Figure 3:**
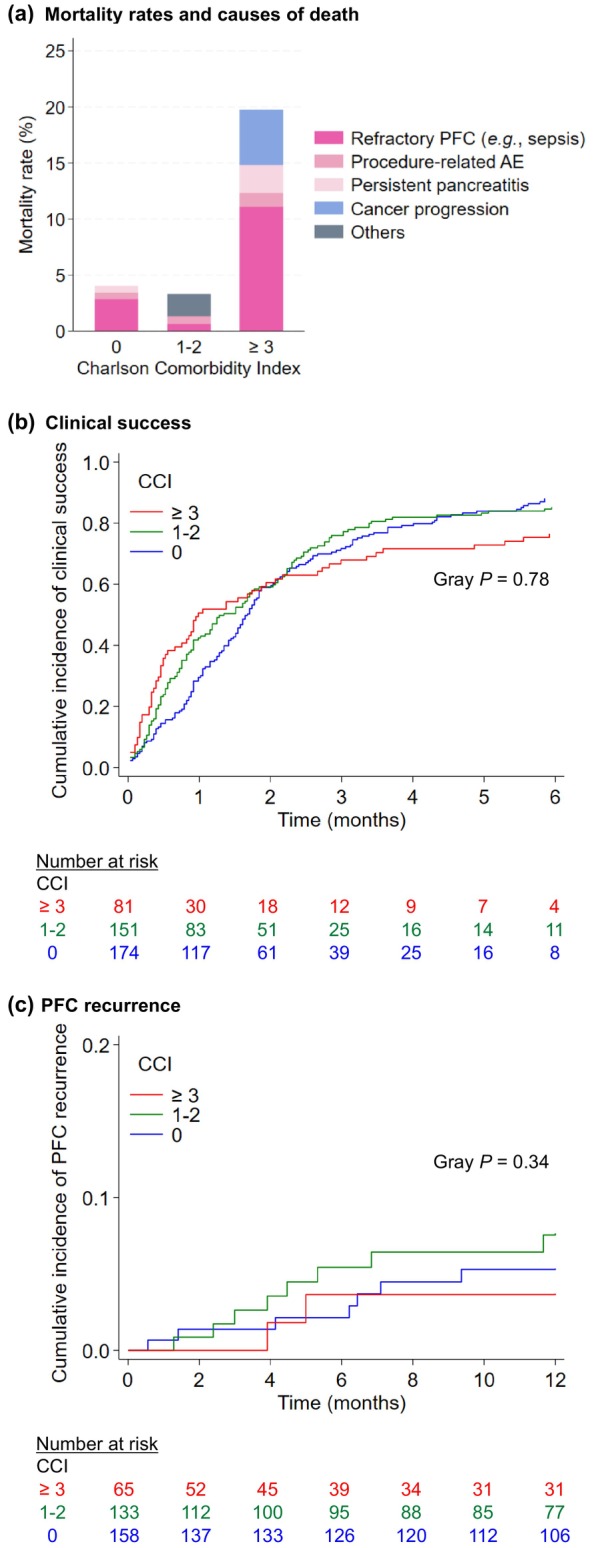
Clinical outcomes of patients receiving endoscopic ultrasound‐guided treatment of pancreatic fluid collections (PFCs) according to Charlson Comorbidity Index (CCI) (a clinical multi‐institutional cohort within the WONDERFUL consortium). (a) Mortality rates and causes of death by CCI. (b) Cumulative incidence curves of clinical success by CCI. (c) Cumulative incidence curves of PFC recurrence by CCI. Given the small numbers of cases and events in the subgroup with CCI of ≥6, the subgroups with CCI of 3–5 and ≥6 were combined for the graphical presentations. AE, adverse event.

In our primary hypothesis testing in the WONDERFUL cohort, higher levels of CCI were associated with a higher rate of in‐hospital mortality (*P*_trend_ < 0.001, Tables 3, S2). Compared to patients with CCI = 0, patients with CCI of 1–2, 3–5, and ≥6 had adjusted ORs of 0.76 (95% CI 0.22–2.54), 5.39 (95% CI 1.74–16.7), and 8.77 (95% CI 2.36–32.6), respectively. The causes of death according to CCI are presented in Figure 3a. In our validation in the DPC cohort, we observed a similar positive association of CCI and the risk of in‐hospital mortality; the adjusted ORs for CCI = 1–2, 3–5, and ≥6 (vs. 0) were 1.21 (95% CI 0.90–1.64), 1.52 (95% CI 0.92–2.49), and 4.84 (95% CI 2.63–8.88), respectively (*P*_trend_ < 0.001). We did not find any effect modification on the CCI‐mortality relationship by the stent types in the WONDERFUL or DPC cohort (*P*_interaction_ > 0.67, Table S3), but the limited sample size of patients treated by a LAMS precluded a robust statistical assessment in this stratum.

**Table 3:** Associations of Charlson Comorbidity Index with clinical outcomes of patients receiving endoscopic ultrasound (EUS)‐guided treatment of pancreatic fluid collections (PFCs)

	Charlson Comorbidity Index				*P*_trend_ ^†^
	0	1–2	3–5	≥6	
WONDERFUL cohort					
*n*	174	151	52	29	‐
In‐hospital mortality					
*n*	7 (4.0%)	5 (3.3%)	10 (19.0%)	6 (21.0%)	‐
Univariable OR (95% CI)	1 (referent)	0.82 (0.25–2.63)	5.68 (2.04–15.8)	6.22 (1.92–20.1)	<0.001
Multivariable OR (95% CI)^‡^	1 (referent)	0.76 (0.22–2.54)	5.39 (1.74–16.7)	8.77 (2.36–32.6)	<0.001
Bleeding					
*n*	12 (6.9%)	13 (8.6%)	6 (12.0%)	2 (6.9%)	‐
Univariable OR (95% CI)	1 (referent)	1.27 (0.56–2.88)	1.76 (0.63–4.95)	1.00 (0.21–4.72)	0.63
Multivariable OR (95% CI)^‡^	1 (referent)	1.39 (0.61–3.20)	2.07 (0.71–6.04)	1.49 (0.30–7.42)	0.37
Length of stay					
Median (IQR), days	41.5 (21–73)	37 (24–67)	40 (23–76)	29 (17–41)	‐
Univariable OR (95% CI)^§^	1 (referent)	0.91 (0.62–1.34)	0.98 (0.56–1.73)	0.52 (0.26–1.04)	0.25
Multivariable OR (95% CI)^‡^ ^,^ ^§^	1 (referent)	0.90 (0.60–1.35)	0.80 (0.44–1.46)	0.53 (0.24–1.16)	0.18
DPC cohort					
*n*	2125	1562	276	90	‐
In‐hospital mortality					
*n*	94 (4.4%)	96 (6.1%)	22 (8.0%)	16 (18.0%)	‐
Univariable OR (95% CI)	1 (referent)	1.41 (1.06–1.90)	1.87 (1.16–3.03)	4.67 (2.62–8.33)	<0.001
Multivariable OR (95% CI)^¶^	1 (referent)	1.21 (0.90–1.64)	1.52 (0.92–2.49)	4.84 (2.63–8.88)	<0.001
Bleeding (hemostatic procedures)					
*n*	121 (5.7%)	87 (5.6%)	17 (6.2%)	6 (6.7%)	‐
Univariable OR (95% CI)	1 (referent)	0.98 (0.74–1.30)	1.09 (0.64–1.84)	1.18 (0.51–2.76)	0.40
Multivariable OR (95% CI)^¶^	1 (referent)	0.98 (0.73–1.30)	1.09 (0.64–1.85)	1.23 (0.52–2.89)	0.35
Length of stay					
Median (IQR), days	22.0 (11–47)	25.0 (12–52)	29.5 (12–57)	36.0 (18–60)	‐
Univariable OR (95% CI)^§^	1 (referent)	1.19 (1.06–1.34)	1.35 (1.08–1.70)	2.16 (1.49–3.12)	<0.001
Multivariable OR (95% CI)^§^ ^,^ ^¶^	1 (referent)	1.16 (1.03–1.30)	1.38 (1.10–1.74)	2.21 (1.51–3.24)	<0.001

^†^ *P*_trend_ was calculated by entering Charlson Comorbidity Index (continuous) in the logistic regression model.

^‡^ The multivariable logistic regression model initially included age (continuous), sex (female vs. male), year of admission (continuous), body mass index (continuous), type of PFC (walled‐off necrosis vs. pseudocyst vs. postoperative PFC), size of PFC (continuous), indication of EUS‐guided drainage (infection vs. abdominal pain vs. expanding PFC vs. others), route of EUS‐guided drainage (transgastric vs. others), and type of stent (plastic vs. metal). Backward elimination with a threshold *P* of 0.10 was conducted to select variables for the final models. The variables that remained in the final models for in‐hospital mortality are described in Table S2.

^§^ Odds ratios (ORs) for a 1‐quartile increase in the length of stay were calculated using the ordinal logistic regression models.

^¶^ The multivariable logistic regression model initially included age (continuous), sex (female vs. male), year of admission (continuous), body mass index (continuous), hospital type (nonacademic vs. academic), hospital case volume (continuous, quartile‐specific medians), and stent type (plastic vs. lumen‐apposing metal stents). Backward elimination with a threshold *P* of 0.10 was conducted to select variables for the final models. The variables that remained in the final model for in‐hospital mortality are described in Table S2.

CI, confidence interval; DPC, Diagnosis Procedure Combination; IQR, interquartile range.

[Correction added on 19 October 2024, after first online publication: A ‘0’ at the end of each *P*‐value has been removed.]

In our secondary analyses (Table 3), CCI was not associated with the risk of bleeding in the WONDERFUL or DPC cohorts (*P*_trend_ > 0.34). In the DPC cohort, higher levels of CCI were associated with longer hospitalization (*P*_trend_ < 0.001). Compared to patients with CCI = 0, patients with CCI of 1–2, 3–5, and ≥6 had adjusted ORs for a unit increase in quartiles of length of stay were 1.16 (95% CI 1.03–1.30), 1.38 (95% CI 1.10–1.74), and 2.21 (95% CI 1.51–3.24), respectively. In contrast, such a positive association was not observed in the WONDERFUL cohort (*P*_trend_ = 0.18). Given the possibility that premature deaths in the CCI‐high group might result in the underestimation of the length of stay, we conducted a sensitivity analysis limited to patients without in‐hospital mortality, which yielded consistent results (Table S4).

We further conducted exploratory analyses of cohort‐specific outcomes (Table 4). In the WONDERFUL cohort, an analysis focusing on PFC‐related mortality yielded similar results to the primary analysis of all‐cause in‐hospital mortality (*P*_trend_ = 0.052), although the association did not reach statistical significance. CCI was not associated with the likelihoods of all procedure‐related adverse events, clinical success, or PFC recurrence (*P*_trend_ > 0.13, Table 4, Fig. 3b,c). In the DPC cohort, CCI was positively associated with total costs for inpatient care (*P*_trend_ < 0.001).

**Table 4:** Associations of Charlson Comorbidity Index with cohort‐specific clinical outcomes of patients receiving endoscopic ultrasound (EUS)‐guided treatment of pancreatic fluid collections (PFCs)

	Charlson Comorbidity Index				*P*_trend_ ^†^
	0	1–2	3–5	≥6	
WONDERFUL cohort					
*n*	174	151	52	29	‐
PFC‐related mortality					
*n*	7 (4.0%)	2 (1.3%)	10 (19.0%)	2 (6.9%)	‐
Univariable OR (95% CI)	1.00 (referent)	0.32 (0.07–1.57)	5.68 (2.04–15.8)	1.77 (0.35–8.96)	0.027
Multivariable OR (95% CI)^‡^	1.00 (referent)	0.35 (0.07–1.71)	6.70 (2.31–19.4)	2.29 (0.43–12.0)	0.052
All procedure‐related adverse events					
*n*	30 (17.0%)	22 (15.0%)	9 (17.0%)	2 (6.9%)	‐
Univariable OR (95% CI)	1.00 (referent)	0.82 (0.45–1.49)	1.00 (0.44–2.28)	0.36 (0.08–1.58)	0.27
Multivariable OR (95% CI)^‡^	1.00 (referent)	0.88 (0.48–1.63)	1.17 (0.50–2.74)	0.48 (0.10–2.18)	0.53
Clinical success					
*n*	158 (91%)	133 (88%)	40 (77%)	25 (86%)	‐
Univariable SHR (95% CI)	1.00 (referent)	1.05 (0.85–1.31)	0.73 (0.51–1.04)	1.46 (0.80–2.66)	0.79
Multivariable SHR (95% CI)^§^	1.00 (referent)	0.97 (0.77–1.22)	0.64 (0.44–0.93)	1.24 (0.70–2.18)	0.67
PFC recurrence^¶^					
*n*	14 (8.9%)	11 (8.3%)	1 (2.5%)	1 (4.0%)	‐
Univariable SHR (95% CI)	1.00 (referent)	0.97 (0.44–2.13)	0.28 (0.04–2.17)	0.46 (0.06–3.56)	0.14
Multivariable SHR (95% CI)^§^	1.00 (referent)	1.15 (0.52–2.52)	0.44 (0.05–3.54)	0.32 (0.03–3.01)	0.14
DPC cohort					
*n*	2125	1562	276	90	‐
Total costs for inpatient care					
Median (IQR), USD	11,491 (6661–23,687)	12,442 (7098–26,039)	14,377 (6852–28,447)	16,587 (10,658–30,745)	‐
Univariable OR (95% CI)^††^	1.00 (referent)	1.13 (1.00–1.27)	1.32 (1.05–1.65)	2.05 (1.42–2.96)	<0.001
Multivariable OR (95% CI)^††^ ^,^ ^‡‡^	1.00 (referent)	1.13 (1.00–1.28)	1.39 (1.10–1.75)	2.21 (1.51–3.24)	<0.001

^†^ *P*_trend_ was calculated by entering Charlson Comorbidity Index (continuous) in the logistic or proportional hazards regression model.

^‡^ The multivariable logistic regression model initially included age (continuous), sex (female vs. male), year of admission (continuous), body mass index (continuous), type of PFC (walled‐off necrosis vs. pseudocyst vs. postoperative PFC), size of PFC (continuous), indication of EUS‐guided drainage (infection vs. abdominal pain vs. expanding PFC vs. others), route of EUS‐guided drainage (transgastric vs. others), and type of stent (plastic vs. metal). Backward elimination with a threshold *P* of 0.10 was conducted to select variables for the final models.

^§^ The multivariable proportional hazards regression model initially included age (continuous), sex (female vs. male), year of admission (continuous), body mass index (continuous), type of PFC (walled‐off necrosis vs. pseudocyst vs. postoperative PFC), size of PFC (continuous), indication of EUS‐guided drainage (infection vs. abdominal pain vs. expanding PFC vs. others), route of EUS‐guided drainage (transgastric vs. others), type of stent (plastic vs. metal), and injury of the pancreatic duct (absent vs. present, only for the model for PFC recurrence^16^). Backward elimination with a threshold *P* of 0.10 was conducted to select variables for the final models. For cases where an injury of the pancreatic duct was inaccessible (1.7%), we assigned a major category and subsequently confirmed that excluding cases with missing data did not alter our findings substantially (data not shown).

^¶^ The analyses of PFC recurrence included 356 cases with clinical success.

^††^ Odds ratios (ORs) for a 1‐quartile increase of total costs were calculated using the ordinal logistic regression models.

^‡‡^ The multivariable logistic regression model initially included age (continuous), sex (female vs. male), year of admission (continuous), body mass index (continuous), hospital type (nonacademic vs. academic), hospital case volume (continuous, quartile‐specific medians), and stent type (plastic vs. lumen‐apposing metal stents). Backward elimination with a threshold *P* of 0.10 was conducted to select variables for the final models.

CI, confidence interval; DPC, Diagnosis Procedure Combination; IQR, interquartile range; SHR, subdistribution hazard ratio; USD, US dollars.

[Correction added on 19 October 2024, after first online publication: A ‘0’ at the end of each *P*‐value has been removed.]

## DISCUSSION

In our clinical multi‐institutional cohort, we have demonstrated that higher levels of CCI are associated with a higher risk of in‐hospital mortality among patients receiving EUS‐guided treatment of PFCs. These findings were validated by utilizing population‐based real‐world data. The higher risk of in‐hospital mortality according to increasing levels of CCI was observed irrespective of the stent types. Prolonged hospitalizations were noted in patients with higher levels of CCI in the population‐based cohort consisting of a large number of hospitals in various settings, but not in our clinical cohort. CCI was not associated with the risk of procedure‐related adverse events. Our data support the potential of CCI as a simple measure to stratify the risk of periprocedural deaths in this population. Prior studies have identified disease and procedural characteristics (e.g., PFC types) as predictive factors for adverse outcomes, and the addition of CCI would refine the risk stratification.^14^, ^15^, ^16^, ^17^ Our findings also highlight the necessity of optimizing treatment strategies for patients with high levels of CCI.

Based on multilevel sources of data, the current study suggests that patients with an increasing number of comorbidities may be at higher risk of fatal outcomes during the periprocedural period of EUS‐guided treatment of PFCs. There are several possible explanations for the negative impact of CCI on clinical outcomes. The tendency of endoscopists to provide less intensive treatment for patients with high levels of CCI may underlie the association. However, in our clinical multicenter cohort, the time to clinical success did not differ by the levels of CCI, and therefore the reluctance of the endoscopists per se might not cause unfavorable outcomes. Supportive care for impaired systemic conditions from sepsis, malnutrition, and so forth, potentially has a substantial impact on the clinical outcomes of EUS‐guided PFC treatment^27^ but may not be administered effectively for patients with a higher burden of comorbidities (e.g., chronic liver and renal failures). In addition, the accumulation of comorbidities (e.g., diabetes and cancer) may be correlated with decreased physiological reserve,^34^, ^35^ which may increase the risk of mortality in case of severe adverse events such as sepsis and perforation of the PFC wall. In our clinical multicenter cohort, higher levels of CCI appeared to be associated with a higher risk of PFC‐related mortality as well as that of overall mortality. Therefore, it is considered that high comorbidity burden may render patients intolerable to interventions following the initial EUS‐guided drainage of PFCs owing to the worsened systemic conditions. Further research is warranted to decipher the biological mechanism through which comorbidity burden can worsen clinical outcomes of patients with endoscopically treated PFCs to develop a new treatment strategy for high‐risk patients. Considering accumulating evidence supporting an inverse association of hospital case volume and the risk of adverse outcomes,^29^, ^36^ the transfer to high‐volume centers may be recommended for patients with high levels of CCI. Given that a fraction of patients with CCI ≥6 in the WONDERFUL cohort died from progressive cancer despite successful management of PFCs, the indication for the treatment should be evaluated prudently for patients with concomitant advanced cancer.

In our secondary analyses, a higher burden of comorbidities was not associated with the risk of procedure‐related bleeding. Bleeding has been a potentially life‐threatening adverse event of EUS‐guided PFC treatment.^12^, ^13^, ^14^ Chronic conditions associated with arteriosclerosis might increase the risk of bleeding during the procedures of endoscopic interventions.^37^, ^38^, ^39^, ^40^, ^41^ In cases with bleeding, transarterial embolization with coiling is often required to achieve hemostasis^12^, ^13^, ^14^ but may not be indicated for patients with substantial comorbidities (e.g., chronic renal failure). Our findings suggest that the risk of bleeding may not be elevated by the accumulation of comorbidities, and hemostasis may be achieved irrespective of the levels of comorbidities. Nonetheless, further investigation is warranted to examine the risk of bleeding, focusing on specific types of comorbidities. CCI was positively associated with the length of hospitalization. The positive association was observed in the population‐based cohort, but not in our clinical cohort derived from tertiary care centers with expertise. These findings also suggest the inverse hospital volume–outcome relationship in patients undergoing EUS‐guided PFC treatment.

The current study has notable strengths, including the large sample size of patients receiving EUS‐guided PFC treatment and the validation via the nationwide database. The data from the clinical cohort with detailed information on the patients, procedures, and outcomes allowed us to adjust for a variety of potential confounders in our multivariable analyses. The validation based on large‐scale data derived from a large number of hospitals with diversity in clinical practices and patient characteristics potentially increased the generalizability of our findings. The current study also has several limitations. First, we could not completely exclude the possibility of unmeasured confounding factors in our multivariable models; nonetheless, we adjusted for a variety of clinical parameters including known risk factors for procedure‐related adverse outcomes,^15^ and the adjustment did not alter the results materially. Second, a vast majority of the study population was Japanese. Given differential risks and severities of chronic diseases by race, our findings on comorbidities should be validated in independent populations with racial diversity.

In conclusion, the current study has suggested that CCI well predicts the risk of in‐hospital mortality associated with EUS‐guided PFC treatment. Given the growing popularity of EUS‐guided interventions in the management of symptomatic PFCs, the current study highlights the importance of assessing the comorbidity status before the treatment. Further research is required to elucidate the appropriate management of patients with specific comorbidity and optimize the treatment selection for patients with high comorbidity burden.

## CONFLICT OF INTEREST

Authors H.I. and Y.N. receive research funding and honoraria from Boston Scientific Japan. M.Ta., T.I., and Y.N. serve as Associate Editors in *Digestive* *Endoscopy*. I.Y. serves as an Associate Editor in *DEN Open*. The other authors declare no conflict of interest for this article.

## FUNDING INFORMATION

This work was supported by grants from the Ministry of Health, Labour, and Welfare, Japan (22AA2003 to K.F. and 23AA2003 and 22AA2003 to H.Y.). T.H. was supported by Japan Society for the Promotion of Science (JSPS) KAKENHI grants (JP19K08362 and JP22H02841) and by grants from Takeda Science Foundation and Daiichi Sankyo Company. The funders had no role in study design, data collection and analysis, the decision to publish, or the preparation of the manuscript.

## ETHICS STATEMENT

Approval of the research protocol by an Institutional Reviewer Board: The research based on the clinical data from the WONDERFUL cohort was approved by the ethics committee at each participating center. The use of the DPC database for the current study was approved by the institutional review board of The University of Tokyo (Tokyo, Japan).

Informed Consent: In the WONDERFUL cohort, written informed consent for the procedure was obtained from all patients, and consent for the use of the retrospective data for research was obtained on an opt‐out basis. In the DPC cohort, the requirement for patient informed consent was waived because of the anonymous nature of the data used.

Registry and the Registration No. of the study/trial: The research based on the clinical data from the WONDERFUL cohort was registered with UMIN‐CTR (registration number UMIN000044130).

Animal Studies: N/A.

## DATA TRANSPARENCY STATEMENT

The deidentified data on a clinical cohort within the WONDERFUL consortium and analytic methods used in the current study will be available from the corresponding authors upon reasonable request. The datasets from the Diagnosis Procedure Combination database analyzed during the current study are not publicly available due to the contracts with the hospitals providing the data.

## Supporting information


**Appendix S1** Participating centers.
**Appendix S2** Endoscopic ultrasound (EUS)‐guided and adjunctive treatment of pancreatic fluid collections (PFCs).
**Appendix S3** Statistical analysis.
**Table S1** Conditions and corresponding scores used to calculate Charlson Comorbidity Index as the summation of all scores.
**Table S2** Association of Charlson Comorbidity Index with in‐hospital mortality of patients receiving endoscopic ultrasound‐guided treatment of pancreatic fluid collections (the final multivariable models).
**Table S3** Association of Charlson Comorbidity Index with in‐hospital mortality of patients receiving endoscopic ultrasound‐guided treatment of pancreatic fluid collections, stratified by stent types.
**Table S4** Association of Charlson Comorbidity Index with the length of stay among patients receiving endoscopic ultrasound‐guided treatment of pancreatic fluid collections without in‐hospital mortality.
