# Supplementary material for: Comorbidity burden and outcomes of endoscopic ultrasound‐guided treatment of pancreatic fluid collections: Multicenter study with nationwide data‐based validation
Source: Dig Endosc. 2024 Sep 26;37(4):413–25. doi: 10.1111/den.14924 (PMC11986896; doi:10.1111/den.14924)
Supplement: Supplementary file 1 — Appendix S1 Participating centers. Appendix S2 Endoscopic ultrasound (EUS)‐guided and adjunctive treatment of pancreatic fluid collections (PFCs). Appendix S3 Statistical analysis. Table S1 Conditions and corresponding scores used to calculate Charlson Comorbidity Index as the summation of all scores. Table S2 Association of Charlson Comorbidity Index with in‐hospital mortality of patients receiving endoscopic ultrasound‐guided treatment of pancreatic fluid collections (the final multivariable models). Table S3 Association of Charlson Comorbidity Index with in‐hospital mortality of patients receiving endoscopic ultrasound‐guided treatment of pancreatic fluid collections, stratified by stent types. Table S4 Association of Charlson Comorbidity Index with the length of stay among patients receiving endoscopic ultrasound‐guided treatment of pancreatic fluid collections without in‐hospital mortality. [file DEN-37-413-s001.docx]

**Appendix S1**

**Participating centers**

The participating centers of the WONDERFUL study group were as follows: Gifu Municipal Hospital, Gifu; Gifu Prefectural General Medical Center, Gifu; Gifu University Hospital, Gifu; Hyogo Medical University Hospital, Hyogo; Juntendo University Hospital, Tokyo; Kindai University Hospital, Osaka; Kobe University Hospital, Hyogo; Saitama Medical Center, Saitama; The University of Tokyo Hospital, Tokyo; and Toyama University Hospital, Toyama (all in Japan).

**Appendix S2**

**Endoscopic ultrasound (EUS)-guided and adjunctive treatment of pancreatic fluid collections (PFCs)**

Despite variations between the participating centers, endoscopic procedures during the study period were standardized to some extent. Contrast-enhanced computed tomography was conducted to evaluate the overall architecture of a PFC as well as levels of PFC encapsulation and a possibility of a pseudoaneurysm located in the puncture route unless there were contraindications. A linear-array echoendoscope was inserted with moderate sedation, and transmural placement of a plastic or metal stent was carried out under endosonographic and fluoroscopic guidance. We used double pigtail plastic stent(s) until a lumen-apposing metal stent (LAMS) with electrocautery enhanced delivery (Hot AXIOS; Boston Scientific Japan, Tokyo, Japan) was approved in October 2018 in Japan and have used the LAMS as a first-choice option thereafter (particularly for cases with walled-off necrosis or large-size pseudocysts). Before the approval of the LAMS, a biflanged metal stent (Niti-S Nagi; Taewoong Medical, Gyeonggi-do, South Korea)[^1^](#_ENREF_1) was used as a clinical trial at some centers. The types of stents were selected at the endoscopist’s discretion and the availability at the center. For patients who were amenable to the initial drainage alone, the step-up treatment was undertaken mainly *via* endoscopic procedures: *i.e.*, endoscopic necrosectomy, additional EUS-guided drainage (referred to as the multigateway approach),[^2^](#_ENREF_2) and/or additional drainage through the initial puncture tract.[^3^](#_ENREF_3) Percutaneous drainage was considered as a step-up approach for PFC lesions apart from the initially targeted PFC and the gastroduodenal lumen. When the symptoms were not managed through those treatment modalities, the patients were referred for salvage surgical interventions (*e.g.*, debridement, drainage, cystogastrostomy).

**Appendix S3**

**Statistical analysis**

Our primary hypothesis testing was an assessment of the association of Charlson Comorbidity Index (CCI) with the in-hospital mortality rate of patients receiving EUS-guided treatment of PFCs in the multivariable logistic regression model. Stratum-specific risk estimates represented secondary analyses. To calculate odds ratios (ORs) and 95% confidence intervals for in-hospital mortality according to CCI, we used the logistic regression model. In the WONDERFUL cohort, the multivariable logistic regression model initially included the following variables to adjust for potential confounding factors: age (continuous), sex (female vs. male), year of admission (continuous), body mass index (continuous), type of PFC (walled-off necrosis vs. pseudocyst vs. postoperative PFC), size of PFC (continuous), indication of EUS-guided drainage (infection vs. abdominal pain vs. expanding PFC vs. others), route of EUS-guided drainage (transgastric vs. others), and type of stent (plastic vs. metal). In the Diagnosis Procedure Combination cohort, the multivariable logistic regression model initially included the following variables: age (continuous), sex (female vs. male), year of admission (continuous), body mass index (continuous), hospital type (non-academic vs. academic), hospital case volume (continuous, quartile-specific medians), and stent type (plastic vs. lumen-apposing metal stents). A backward elimination with a threshold *P* of 0.10 was conducted to select variables for the final models. Multivariable original logistic regression models were used for analyses of quartiles of the length of stay and total costs as outcome variables. For cases with missing data on body mass index (2.0% and 4.4% in the WONDERFUL and Diagnosis Procedure Combination cohorts, respectively), we assigned the median value and subsequently confirmed that excluding cases with missing data did not alter our findings substantially (data not shown). A sensitivity analysis excluding non-LAMS metal stents in the WONDERFUL cohort did not alter our findings substantially (data not shown). In analyses of in-hospital deaths stratified by stent types, a statistical interaction was assessed by using the Wald test on the cross-product of CCI (continuous) and stent types (plastic stent vs. LAMS) in the logistic regression model. The stratum-specific ORs were computed based on a single regression model with a re-parameterization of an interaction term.[^4^](#_ENREF_4) In secondary analyses, cumulative incidence functions of clinical success and PFC recurrence were estimated *via* the competing risk framework, in which competing risk events included salvage surgery or death for clinical success and death for PFC recurrence.[^5^](#_ENREF_5)^,^[^6^](#_ENREF_6) In this framework, time to clinical success was defined as the time from the initial EUS-guided drainage to clinical success, the last follow-up, or death, whichever came first. Time to PFC recurrence was defined as the time from clinical success to PFC recurrence, the last follow-up, or death, whichever came first. Patients who lost to follow-up were dealt with as censored cases at the time of the last follow-up. Cumulative incidence functions were compared between groups *via* Gray’s test using the SAS software (version 9.4; SAS Institute, Cary, NC, USA). To calculate subdistribution hazard ratios and 95% confidence intervals, we used the proportional hazards regression model for competing risks. To adjustment for potential confounding factors, the multivariable proportional hazards regression model initially included the same set of covariates in the multivariable logistic regression model as well as injury of the pancreatic duct (absent vs. present) for the model for PFC recurrence. The assumption of proportional hazards was generally satisfied by assessing a time-dependent covariate, which was the cross-product of CCI and time to clinical success or PFC recurrence (*P* > 0.05).

To compare characteristics between the CCI subgroups, we used the chi-square test for categorical variables and the analysis of variance or the Kruskal-Wallis test, as appropriate, for continuous variables.

All statistical analyses were performed using the Stata software (version 18, StataCorp LLC, College Station, Texas, USA), unless otherwise noted, and all *P* values were two-sided. Given multiple comparisons, we used the stringent two-sided α level of 0.005 for statistical significance.[^7^](#_ENREF_7)

**References**

1 Yamamoto N, Isayama H, Kawakami H *et al.* Preliminary report on a new, fully covered, metal stent designed for the treatment of pancreatic fluid collections. *Gastrointest Endosc* 2013; **77**: 809-14.

2 Varadarajulu S, Phadnis MA, Christein JD, Wilcox CM. Multiple transluminal gateway technique for eus-guided drainage of symptomatic walled-off pancreatic necrosis. *Gastrointest Endosc* 2011; **74**: 74-80.

3 Mukai S, Itoi T, Sofuni A *et al.* Novel single transluminal gateway transcystic multiple drainages after eus-guided drainage for complicated multilocular walled-off necrosis (with videos). *Gastrointest Endosc* 2014; **79**: 531-5.

4 Hamada T, Cao Y, Qian ZR *et al.* Aspirin use and colorectal cancer survival according to tumor cd274 (programmed cell death 1 ligand 1) expression status. *J Clin Oncol* 2017; **35**: 1836-44.

5 Marubini E, Valsecchi MG. Analysing survival data from clinical trials and observational studies. *JohnWiley and Sons, NewYork* 1995.

6 Hamada T, Nakai Y, Isayama H *et al.* Estimation and comparison of cumulative incidences of biliary self-expandable metallic stent dysfunction accounting for competing risks. *Dig Endosc* 2014; **26**: 270-5.

7 Benjamin DJ, Berger JO, Johannesson M *et al.* Redefine statistical significance. *Nature Human Behaviour* 2018; **2**: 6-10.

Table S1. Conditions and corresponding scores used to calculate Charlson Comorbidity Index as the summation of all scores

| Conditions | Scores |
| --- | --- |
| Myocardial infarction | 1 |
| Congestive heart failure | 1 |
| Peripheral vascular disease | 1 |
| Cerebrovascular disease | 1 |
| Dementia | 1 |
| Chronic pulmonary disease | 1 |
| Connective tissue disease | 1 |
| Ulcer disease | 1 |
| Mild liver disease | 1 |
| Diabetes without end-organ damage | 1 |
| Hemiplegia | 2 |
| Moderate or severe renal disease | 2 |
| Diabetes with end organ damage | 2 |
| Any tumor without metastasis | 2 |
| Leukemia | 2 |
| Lymphoma | 2 |
| Moderate or severe liver disease | 3 |
| Metastatic solid tumor | 6 |
| Acquired immune deficiency syndrome | 6 |
|  |  |

Table S2. Association of Charlson Comorbidity Index with in-hospital mortality of patients receiving endoscopic ultrasound-guided treatment of pancreatic fluid collections (the final multivariable models)

|  | Multivariable OR (95% CI)^†^  for in-hospital mortality |
| --- | --- |
| **WONDERFUL cohort** |  |
| Charlson Comorbidity Index |  |
| 0 | 1 (referent) |
| 1-2 | 0.76 (0.22-2.54) |
| 3-5 | 5.39 (1.74-16.7) |
| ≥ 6 | 8.77 (2.36-32.6) |
|  |  |
| Age (per 10-year increase) | 1.49 (1.02-2.16) |
|  |  |
| Year of admission (per 5-year increase) | 0.58 (0.33-1.02) |
|  |  |
| Size of PFC (per 5-cm increase) | 2.20 (1.45-3.33) |
|  |  |
| **DPC cohort** |  |
| Charlson Comorbidity Index |  |
| 0 | 1 (referent) |
| 1-2 | 1.21 (0.90-1.64) |
| 3-5 | 1.52 (0.92-2.49) |
| ≥ 6 | 4.84 (2.63-8.88) |
|  |  |
| Age (per 10-year increase) | 1.53 (1.36-1.71) |
|  |  |
| Year of admission (per 5-year increase) | 0.70 (0.53-0.94) |
|  |  |
| Body mass index (per 5-kg/m^2^ increase) | 1.65 (1.40-1.95) |
|  |  |
| Hospital case volume |  |
| Q1 (1.0-1.9 per year) | 1 (referent) |
| Q2 (2.0-3.4 per year) | 0.98 (0.70-1.37) |
| Q3 (3.5-7.9 per year) | 0.51 (0.35-0.75) |
| Q4 (≥ 8.0 per year) | 0.28 (0.17-0.46) |
|  |  |
| Stent type |  |
| Plastic stent | 1 (referent) |
| LAMS | 2.87 (1.84-4.48) |
|  |  |

^†^ In the WONDERFUL cohort, the multivariable logistic regression model initially included age (continuous), sex (female vs. male), year of admission (continuous), body mass index (continuous), type of PFC (walled-off necrosis vs. pseudocyst vs. postoperative PFC), size of PFC (continuous), indication of EUS-guided drainage (infection vs. abdominal pain vs. expanding PFC vs. others), route of EUS-guided drainage (transgastric vs. others), and type of stent (plastic vs. metal). In the DPC cohort, the multivariable logistic regression model initially included age (continuous), sex (female vs. male), year of admission (continuous), body mass index (continuous), hospital type (non-academic vs. academic), hospital case volume (continuous, quartile-specific medians), and stent type (plastic vs. LAMS). Backward elimination with a threshold *P* of 0.10 was conducted to select variables for the final models.

Abbreviations: CI, confidence interval; DPC, Diagnosis Procedure Combination; EUS, endoscopic ultrasound; LAMS, lumen-apposing metal stent; OR, odds ratio; PFC, pancreatic fluid collection; Q1-4, quartiles 1-4.

Table S3. Association of Charlson Comorbidity Index with in-hospital mortality of patients receiving endoscopic ultrasound-guided treatment of pancreatic fluid collections, stratified by stent types

|  | Charlson Comorbidity Index | | | |  |  |
| --- | --- | --- | --- | --- | --- | --- |
|  | 0 | 1-2 | 3-5 | ≥ 6 | *P*_trend_^§^ | *P*_interaction_^¶^ |
| **WONDERFUL cohort** |  |  |  |  |  |  |
| **Plastic stent** |  |  |  |  |  |  |
| No. of cases (n = 290) | 122 | 108 | 37 | 23 |  |  |
| No. of in-hospital deaths | 6 (4.9%) | 3 (2.8%) | 5 (14%) | 6 (26%) |  |  |
| Univariable OR (95% CI) | 1 (referent) | 0.55 (0.13-2.26) | 3.02 (0.87-10.5) | 6.82 (1.97-23.6) | < 0.001 | 0.90 |
| Multivariable OR (95% CI)^†^ | 1 (referent) | 0.53 (0.12-2.27) | 2.62 (0.69-10.0) | 6.57 (1.74-24.9) | 0.001 | 0.74 |
|  |  |  |  |  |  |  |
| **Metal stent (including LAMS)** |  |  |  |  |  |  |
| No. of cases (n = 116) | 52 | 43 | 15 | 6 |  |  |
| No. of in-hospital deaths | 1 (1.9%) | 2 (4.7%) | 5 (33%) | 0 |  |  |
| Univariable OR (95% CI) | 1 (referent) | 2.49 (0.22-28.4) | 25.5 (2.68-242) | NA | 0.060 |  |
| Multivariable OR (95% CI)^†^ | 1 (referent) | 2.25 (0.19-26.5) | 21.3 (2.10-216) | NA | 0.035 |  |
|  |  |  |  |  |  |  |
| **DPC cohort** |  |  |  |  |  |  |
| **Plastic stent** |  |  |  |  |  |  |
| No. of cases (n = 3,647) | 1,898 | 1,420 | 248 | 81 |  |  |
| No. of in-hospital deaths | 74 (3.9%) | 78 (5.5%) | 17 (6.9%) | 15 (19%) |  |  |
| Univariable OR (95% CI) | 1 (referent) | 1.43 (1.03-1.98) | 1.81 (1.05-3.13) | 5.60 (3.05-10.3) | < 0.001 | 0.45 |
| Multivariable OR (95% CI)^‡^ | 1 (referent) | 1.20 (0.86-1.67) | 1.42 (0.81-2.47) | 5.40 (2.86-10.2) | < 0.001 | 0.68 |
|  |  |  |  |  |  |  |
| **LAMS** |  |  |  |  |  |  |
| No. of cases (n = 406) | 227 | 142 | 28 | 9 |  |  |
| No. of in-hospital deaths | 20 (8.8%) | 18 (13%) | 5 (18%) | 1 (11%) |  |  |
| Univariable OR (95% CI) | 1 (referent) | 1.50 (0.77-2.95) | 2.25 (0.77-6.56) | 1.29 (0.15-10.9) | 0.14 |  |
| Multivariable OR (95% CI)^‡^ | 1 (referent) | 1.29 (0.64-2.60) | 2.03 (0.67-6.14) | 1.71 (0.19-15.3) | 0.16 |  |
|  |  |  |  |  |  |  |

^†^ The multivariable logistic regression model initially included age (continuous), sex (female vs. male), year of admission (continuous), body mass index (continuous), type of PFC (walled-off necrosis vs. pseudocyst vs. postoperative PFC), size of PFC (continuous), indication of EUS-guided drainage (infection vs. abdominal pain vs. expanding PFC vs. others), and route of EUS-guided drainage (transgastric vs. others). Backward elimination with a threshold *P* of 0.10 was conducted to select variables for the final models.

^‡^ The multivariable logistic regression model initially included age (continuous), sex (female vs. male), year of admission (continuous), body mass index (continuous), hospital type (non-academic vs. academic), and hospital case volume (continuous, quartile-specific medians). Backward elimination with a threshold *P* of 0.10 was conducted to select variables for the final models.

^§^ *P*_trend_ was calculated by entering Charlson Comorbidity Index (continuous) in the logistic regression model.

^¶^ *P*_interaction_ was calculated by evaluating the Wald test for a cross-product term of Charlson Comorbidity Index (continuous) and stent type (plastic vs. metal) in the logistic regression model.

Abbreviations: CI, confidence interval; DPC, Diagnosis Procedure Combination; EUS, endoscopic ultrasound; LAMS, lumen-apposing metal stent; OR, odds ratio; PFC, pancreatic fluid collection.

Table S4. Association of Charlson Comorbidity Index with the length of stay among patients receiving endoscopic ultrasound-guided treatment of pancreatic fluid collections without in-hospital mortality

|  | Charlson Comorbidity Index | | | |  |
| --- | --- | --- | --- | --- | --- |
|  | 0 | 1-2 | 3-5 | ≥ 6 | *P*_trend_^¶^ |
| **WONDERFUL cohort** |  |  |  |  |  |
| Length of stay |  |  |  |  |  |
| n | 167 | 146 | 42 | 23 |  |
| Median (IQR), days | 41 (20-73) | 37 (24-66) | 46 (23-79) | 27 (15-36) |  |
| Univariable OR (95% CI)^‡^ | 1 (referent) | 0.93 (0.63-1.39) | 1.12 (0.60-2.09) | 0.43 (0.20-0.94) | 0.24 |
| Multivariable OR (95% CI)^†‡^ | 1 (referent) | 0.91 (0.60-1.37) | 0.91 (0.47-1.76) | 0.46 (0.19-1.12) | 0.21 |
|  |  |  |  |  |  |
| **DPC cohort** |  |  |  |  |  |
| Length of stay |  |  |  |  |  |
| n | 2,031 | 1,466 | 254 | 74 |  |
| Median (IQR), days | 21 (10-44) | 23 (11-48) | 26 (11-51) | 32.5 (16-60) |  |
| Univariable OR (95% CI)^‡^ | 1 (referent) | 1.18 (1.05-1.33) | 1.26 (1.00-1.60) | 2.03 (1.35-3.05) | < 0.001 |
| Multivariable OR (95% CI)^‡§^ | 1 (referent) | 1.17 (1.03-1.32) | 1.34 (1.05-1.71) | 2.15 (1.41-3.28) | < 0.001 |
|  |  |  |  |  |  |

^†^ The multivariable logistic regression model initially included age (continuous), sex (female vs. male), year of admission (continuous), body mass index (continuous), type of PFC (walled-off necrosis vs. pseudocyst vs. postoperative PFC), size of PFC (continuous), indication of EUS-guided drainage (infection vs. abdominal pain vs. expanding PFC vs. others), route of EUS-guided drainage (transgastric vs. others), and type of stent (plastic vs. metal). Backward elimination with a threshold *P* of 0.10 was conducted to select variables for the final models.

^‡^ ORs for a 1-quartile increase in the length of stay were calculated using the ordinal logistic regression models.

^§^ The multivariable logistic regression model initially included age (continuous), sex (female vs. male), year of admission (continuous), body mass index (continuous), hospital type (non-academic vs. academic), hospital case volume (continuous, quartile-specific medians), and stent type (plastic vs. lumen-apposing metal stents). Backward elimination with a threshold *P* of 0.10 was conducted to select variables for the final models.

^¶^ *P*_trend_ was calculated by entering Charlson Comorbidity Index (continuous) in the logistic regression model.

Abbreviations: CI, confidence interval; DPC, Diagnosis Procedure Combination; EUS, endoscopic ultrasound; IQR, interquartile range; OR, odds ratio; PFC, pancreatic fluid collection.
